# Shame and psychopathology. Its role in the genesis and perpetuation of different disorders

**DOI:** 10.1192/j.eurpsy.2022.1811

**Published:** 2022-09-01

**Authors:** F. Azevedo, R. André, A. Quintão, D. Jeremias, C. Almeida

**Affiliations:** 1 Centro Hospitalar de Lisboa Ocidental, Psychiatry, Lisbon, Portugal; 2 Centro Hospitalar Universitário Lisboa Norte, Psychiatry, Lisboa, Portugal; 3 Centro Hospitalar de Lisboa Ocidental, Psychiatry Department, Lisboa, Portugal; 4 NOVA Medical School, Psychiatry And Mental Health, Lisboa, Portugal

**Keywords:** Psychopathology, Disorders, shame

## Abstract

**Introduction:**

Shame is a profound negative emotion that can sometimes be cover up by guilt and remain undiagnosed. Shame and guilt have been described as self-conscious and moral emotions as they both involve self-evaluation and lay a role in facilitating moral conduct. They derive from the notion of responsibility, but some authors suggest that while guilt focuses only on the act at hand shame focuses on the one executing it. The self is the object.

**Objectives:**

To review the literature on shame and its role in different disorders both as a causing agent and as a perpetuating agent

**Methods:**

Non-systematic review of the literature with selection of scientific articles published in the past 20 years; by searching Pubmed and Medscape databases using the combination of MeSH descriptors. The following MeSH terms were used: “shame”, “psychopathology.

**Results:**

Since shame globally decreases self-esteem and is an awareness of personal flaws it can lead to the feelings of helplessness and the development or worsening of mental disorders. As such it is no wonder to find shame being studied in many different forms, more and less structured with important connections being made with social anxiety, eating disorders, dysmorphic disorders, personality disorders and bereavement.

**Conclusions:**

Shame’s role, independently from guilt can have an impact on both the genesis and perpetuation of mental disorders. Its study can uncover missing links between different types of experiences and the pathological reactions that may subsequently follow.

**Disclosure:**

No significant relationships.

